# Adhesion, Proliferation and Migration of NIH/3T3 Cells on Modified Polyaniline Surfaces

**DOI:** 10.3390/ijms17091439

**Published:** 2016-09-15

**Authors:** Petra Rejmontová, Zdenka Capáková, Nikola Mikušová, Nela Maráková, Věra Kašpárková, Marián Lehocký, Petr Humpolíček

**Affiliations:** Centre of Polymer Systems, Tomas Bata University in Zlín, třída Tomáše Bati 5678, 760 01 Zlín, Czech Republic; rejmontova@cps.utb.cz (P.R.); kucekova@cps.utb.cz (Z.C.); mikusova@ft.utb.cz (N.M.); marakova@ft.utb.cz (N.M.); vkasparkova@ft.utb.cz (V.K.); lehocky@ft.utb.cz (M.L.)

**Keywords:** polyaniline, fibroblast, cyto-compatibility, sulfamic acid, phosphotungstic acid, poly (2-acrylamido-2-methyl-1-propanesulfonic) acid

## Abstract

Polyaniline shows great potential and promises wide application in the biomedical field thanks to its intrinsic conductivity and material properties, which closely resemble natural tissues. Surface properties are crucial, as these predetermine any interaction with biological fluids, proteins and cells. An advantage of polyaniline is the simple modification of its surface, e.g., by using various dopant acids. An investigation was made into the adhesion, proliferation and migration of mouse embryonic fibroblasts on pristine polyaniline films and films doped with sulfamic and phosphotungstic acids. In addition, polyaniline films supplemented with poly (2-acrylamido-2-methyl-1-propanesulfonic) acid at various ratios were tested. Results showed that the NIH/3T3 cell line was able to adhere, proliferate and migrate on the pristine polyaniline films as well as those films doped with sulfamic and phosphotungstic acids; thus, utilization of said forms in biomedicine appears promising. Nevertheless, incorporating poly (2-acrylamido-2-methyl-1-propanesulfonic) acid altered the surface properties of the polyaniline films and significantly affected cell behavior. In order to reveal the crucial factor influencing the surface/cell interaction, cell behavior is discussed in the context of the surface energy of individual samples. It was clearly demonstrated that the lesser the difference between the surface energy of the sample and cell, the more cyto-compatible the surface is.

## 1. Introduction

Recently, a rise in interest has been shown in research on polyaniline (PANI), which represents a highly attractive material due to its intrinsic conductivity, simple and inexpensive synthesis, versatile surface properties and favorable biological properties. As a direct consequence of such unique properties, PANI exhibits potential for diverse applications—ranging from microelectronics [1] on through biosensors [2] to tissue engineering [3]. Moreover, PANI, as representative of conducting polymers, has the application potentials as micro-electrodes for cell stimulation embedded in the bio-hybrid actuators [4] or material mimicking neuronal activity and neuromorphic functionalities [5]. In terms of biomedicine, a promising PANI electroactive scaffold could be utilized specifically in cardiac or neuronal tissue engineering [6]. However, utilizing PANI in biosensing and tissue engineering assumes that substantial knowledge exists on its bio-interface properties. In this context, a number of studies have already been published, e.g., PANI films doped with perchloride, hydrochloride, malic and citric acid are suitable for adhesion and proliferation of PC-12 [7], PANI films deposited with poly (2-acrylamido-2-methyl-1-propanesulfonic) acid (PAMPSA) allow an embryonic stem cell to adhere and grow [8]. Therefore, polyaniline coating has been used in the past to improve the physical and biological properties of materials such as polyurethane [9] or graphene and graphene oxide [10]. Moreover, not only the modification of the cell/PANI surface interaction is possible, but also the interaction with blood can be influenced. The anticoagulation effect of PANI reprotonated with PAMPSA via the interaction of film with coagulation factors X, V and II has previously been described [11]. Apart from PAMPSA, other acids may be utilized, e.g., sulfamic and phosphotungstic acid.

Thus, the aim herein is to reveal the biological properties of PANI films synthesized by chemical oxidation in pristine forms as well as modified with poly (2-acrylamido-2-methyl-1-propanesulfonic), sulfamic and phosphotungstic acid. The mouse embryonic fibroblast cell line NIH/3T3 was utilized for this purpose. This cell line is generally considered as a suitable cell model for studying material biocompatibility and was already used for the determination of biological properties of conducting polymers [12,13].

## 2. Results and Discussion

### 2.1. Surface Energy

In the case of the evaluation of surface properties and related interactions of NIH/3T3 cells with the studied surfaces, the surface energy of the tested samples as well as the cell monolayer was determined. The total surface energy (γ^tot^) was obtained and the absolute value of the difference between the surface energy of the cells and the sample (γ^dif^) was calculated (Table 1).

An interesting phenomena was observed for polyaniline samples that contained PAMPSA in the reaction mixture. As previously published, a significant decrease in γ^tot^ occurred when pristine polyaniline substrate was doped with PAMPSA [14]. In the present study, in the case of PANI-PAMPSA-1:1, the values obtained for the surface energy corresponded to PANI doped with PAMPSA. This may indicate that the characteristics of PAMPSA predominated and significantly impacted the surface properties of PANI-PAMPSA-1:1. Surface energy changed dramatically when a reduced amount of PAMPSA was used during polyaniline synthesis (the ratio of aniline hydrochloride to PAMPSA for synthesis was 2:1). In this case, the results approximated values for γ^tot^ as observed for pristine PANI-S and PANI-B. Thus, the surface properties of PANI-PAMPSA-2:1 are primarily governed by polyaniline, and only to a lesser extent by PAMPSA. Moreover, as can be seen, doping PANI with sulfamic acid and phosphotungstic acid did not influence surface properties in terms of γ^tot^ in comparison with PANI-S and PANI-B. Furthermore, the obtained γ^tot^ resembled that for the cell monolayer and so should indicate that suitable biological properties exist.

### 2.2. Cyto-Compatibility

**Figure 1:**
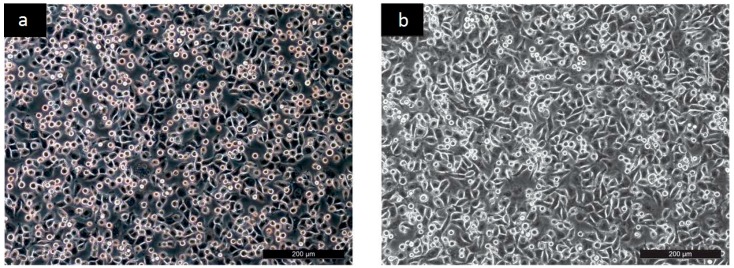
Adhesion: (**a**) Reference; (**b**) PANI-B.

**Figure 2:**
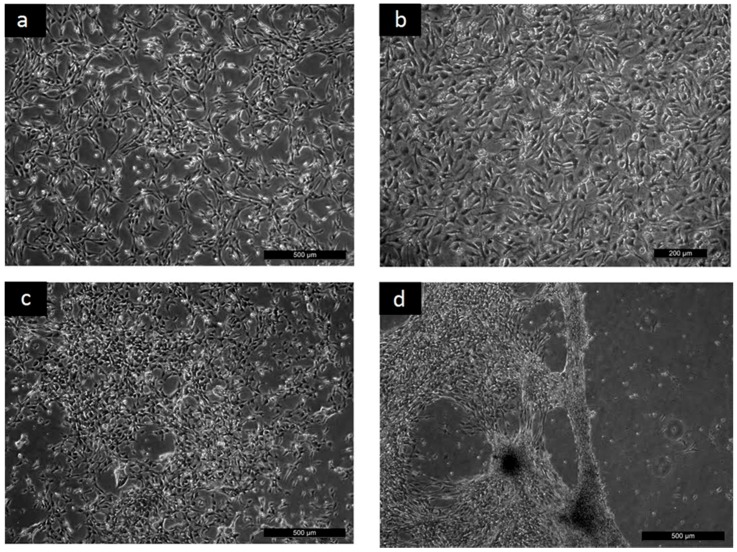
Proliferation: (**a**) Reference 24 h; (**b**) PANI-S 24 h; (**c**) PANI-PAMPSA-1:1 144 h; (**d**) PANI-PAMPSA-2:1 144 h.

To reveal the cyto-compatibility of tested samples, the adhesion, proliferation and migration of the mouse embryonic fibroblast NIH/3T3 cell line was investigated. The NIH/3T3 cell line is one of the most frequently used lines in material/cell interaction research and has been previously used for cytotoxicity testing of PANI [15,16,17]. Thus, the results provided by these tests can easily be compared with data published in the literature. The micrographs clearly show that although the NIH/3T3 cells were able to adhere to all the tested surfaces in a similar way as the reference (Figure 1, only PANI-S is presented), remarkable differences in the subsequent cell proliferation and morphology existed (see Figure 2).

It was clearly demonstrated that after 24 h, the cells reached semi-confluence on the reference sample (Figure 2a). Behavior comparable to that for the reference was observed on the surfaces of PANI-S, PANI-B, PANI-SULF, and PANI-PT (see Figure 2b, where PANI-S is presented as an example). In contrast, on the PANI-PAMPSA-1:1 sample, proliferation was significantly decreased and the cells initially reached a semi-confluent state after 144 h (see Figure 2c). Cell proliferation then improved on PANI-PAMPSA-2:1, which contained a lower amount of PAMSPA compared to PANI-PAMPSA-1:1. This also corresponded to the better cell proliferation observed on pristine PANI-S and PANI-B surfaces, where PAMPSA was absent. Nevertheless, attachment was weak and any cells adhered on PANI-PAMPSA-2:1 easily detached from its surface; even gentle handling during media exchange caused cell detachment (Figure 2d). Consequently, it can be concluded that introducing PAMPSA into the polymer bulk during synthesis notably affected NIH/3T3 cell proliferation in comparison with reference and pristine polyaniline. These results correspond to observing the preferable behavior of mouse embryonic stem cells on pristine PANI forms, in comparison with PANI deposited with PAMPSA [14].

**Figure 3:**
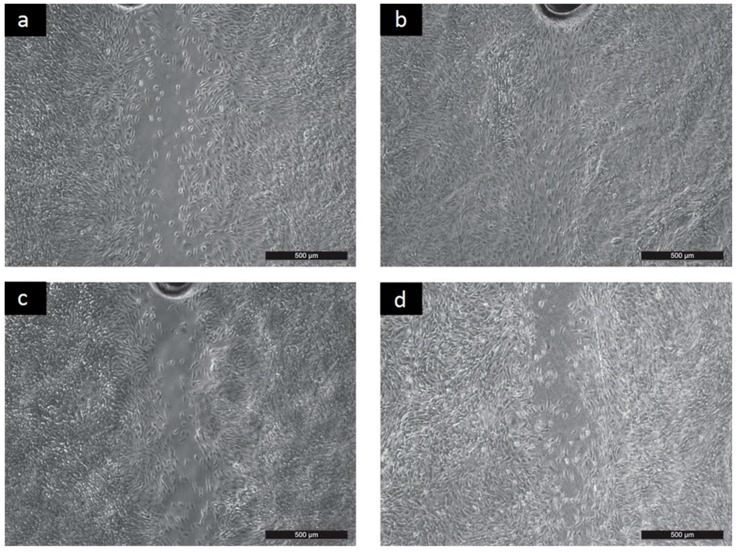
Migration 48 h: (**a**) Reference; (**b**) PANI-B; (**c**) PANI-PAMPSA-1:1; (**d**) PANI-SULF.

The results of cell migration on the tested surfaces are presented in Figure 3.

As can be seen, migration on PANI-PAMPSA surfaces was significantly less than for the reference (Figure 3c, only PANI-PAMPSA-1:1 is given as an example). This finding fully corresponds with the limited cell proliferation observed on these surfaces, and correlates with the results obtained for the surface energy. Obtained results indicate utilization of this modification in the field of biosensors rather than biomaterials. Nevertheless, the migration of cells on PANI-S, PANI-B, PANI-SULF and PANI-PT was comparable to cell behavior on the reference sample (see PANI-B in Figure 3b and PANI-SULF in Figure 3d). In summary, cell behavior corresponds to the surface energy of individual samples. It can be concluded that the less variation there is in the surface energy of a tested sample, the more compatible the surface is. Overall, these findings supported cell behavior in terms of adhesion and proliferation, suggesting the potential of applying such PANI forms in biomedicine, e.g., tissue engineering of electrically responsive tissues.

## 3. Materials and Methods

### 3.1. Preparation of Polyaniline Films

The PANI films were formed in situ directly on the tissue culture plates (TPP, Trasadingen, Switzerland). The polyaniline salt (PANI-S) was prepared by chemical oxidation of aniline hydrochloride with ammonium peroxydisulfate in aqueous solution according to IUPAC technical report [18]. An appropriate amount of aniline hydrochloride (2.59 g, Neratovice, Czech Republic) and ammonium peroxydisulfate (5.71 g, Sigma-Aldrich, St. Louis, MO, USA) was separately dissolved in 50 mL of water. Both solutions were mixed, briefly stirred and poured into culture plates. The polymerization reaction lasted 1 h at room temperature. Then, the solution was poured out and deposited green film of PANI-S was rinsed with 0.2 M hydrochloric acid followed by methanol. The films were left to dry in air overnight.

To prepare polyaniline base (PANI-B), the films were immersed in 1 M ammonium hydroxide for 12 h.

In order to prepare films doped with sulfamic acid (PANI-SULF) and phosphotungstic acid (PANI-PT), the PANI-B films were re-protonated with either 1 M sulfamic acid (Sigma-Aldrich, St. Louis, MO, USA) or 50 wt % aqueous solution of phosphotungstic acid (Sigma-Aldrich, St. Louis, MO, USA). The solutions of acids were poured onto the surface of the PANI-B film and the reaction was left to proceed for 24 h. Afterwards, the residual solutions were poured out and the films were rinsed with methanol and left to dry in air.

The second type of modified film was prepared using direct polymerization with PAMPSA present in the reaction mixture of aniline hydrochloride and ammonium peroxydisulfate. To this end, modified procedures published by Bayer et al. [19], Stejskal et al. [20], and Yoo et al. [21] were employed. Firstly, an aqueous solution of PAMPSA was prepared, its target concentration corresponding to 0.028 mol of its monomer, acrylamido-2-methyl-propanesulfonic acid. Aniline hydrochloride (0.028 mol) was then added to the PAMPSA solution and stirred at room temperature for 1 h. The mole ratio of aniline hydrochloride to PAMPSA was adjusted to 1:1 (PANI-PAMPSA-1:1) or 2:1 (PANI-PAMPSA-2:1). The oxidant, ammonium peroxydisulfate (0.025 mol), was dissolved separately in ultrapure water and added to the reaction mixture at the mole ratio for aniline hydrochloride to oxidizing agent of 1:0.9. Polymerization was completed within 60 min. The films were rinsed with water and methanol to remove any adherent PAMPSA, and left to dry in air.

### 3.2. Surface Energy

The surface energy evaluation system (“SEE System”, Advex Instruments, Brno, Czech Republic) was used to measure contact angle and determine surface energy of tested samples. Deionized water, ethylene glycol and diiodomethane (Sigma-Aldrich, St. Louis, MO, USA) in volume of 2 μL was utilized as testing liquids for PANI films. To obtain the value of contact angle, ten separate readings were performed for each testing liquid. The surface energy was determined by the “acid-base” method.

In order to evaluate the interaction of NIH/3T3 cells with the studied surfaces, the surface energy of the cells was determined. The cell suspension was thoroughly filtered through a filtration paper to obtain a homogeneous cell layer on the paper and this sample was immediately subjected to contact angle measurement. For the cells, glycerol (Sigma-Aldrich, St. Louis, MO, USA) and diiodomethane were used as the testing liquids, and the droplet volume of these liquids was also set to 2 μL for all experiments. In a corresponding manner to the aforementioned polyaniline samples, ten separate readings were taken to obtain one representative average value for the contact angle. Using these data, the surface energy of the cells was calculated by the “OWRK” (Owens-Wendt-Rabel-Kaelble) method, in addition to obtaining total surface energy (γ^tot^) including its components, disperse part (γ^LW^) and polar part (γ^AB^). Finally, calculation was made of γ^dif^, denoting the absolute value of the difference between the surface energy of the cells and sample, according to equation γ^dif^ = |γ^tot,cell^ − γ^tot,sample^|.

### 3.3. Cyto-Compatibility

Prior to in vitro testing, the samples were disinfected by 30 min of exposure to a UV-radiation source operating at a wavelength of 258 nm, emitted by a low-pressure mercury lamp. Investigation was conducted into the compatibility of the polyaniline films with the mouse embryonic fibroblast NIH/3T3 cell line (ATCC CRL-1658 NIH/3T3, Marlboro, MA, USA). NIH/3T3 cell line is abundantly used in material biocompatibility testing, thus obtained results are readily comparable to published data in literature. Definitively, study was made of cell adhesion, proliferation and migration. ATCC-formulated, Dulbecco’s Modified Eagle’s Medium (PAA, Trasadingen, Switzerland) containing 10% calf serum (PAA, Trasadingen, Switzerland) and 100 U·mL^−1^ Penicillin/Streptomycin (PAA, Trasadingen, Switzerland) was used as the culture medium.

Tests were conducted as follows:

1. To reveal the ability of cells to adhere to the surfaces, the cells were seeded on reference culture dishes (TPP, Trasadingen, Switzerland) and the studied polymer films at a concentration of 1 × 10^7^ cells·mL^−1^. After one hour, the cells were gently rinsed and micrographs were taken.
2. Cell proliferation and morphology were evaluated on cells that had been seeded at an initial concentration of 1 × 10^5^ cells·mL^−1^ and cultivated.
3. Cell migration was determined by the scratch assay according to Liang et al. [22] with modification. The scratch assay was created in a confluent cell monolayer. After 48 h had passed, micrographs were captured with an Olympus inverted fluorescent microscope (Olympus, IX51, Tokyo, Japan) equipped with a digital color camera (Leica DFC480, Wetzlar, Germany).

## 4. Conclusions

The cell/surface interaction of pristine polyaniline films, films doped with sulfamic and phosphotungstic acid and films incorporating poly (2-acrylamido-2-methyl-1-propanesulfonic) were studied. The mouse embryonic fibroblasts were able to adhere, proliferate and migrate on pristine polyaniline films and those doped with sulfamic or phosphotungstic acids. Therefore, these polyaniline forms should be suitable for utilization in biomedicine, e.g., tissue engineering of electrically responsive tissues. Contrarily, incorporating poly (2-acrylamido-2-methyl-1-propanesulfonic) acid actually affected the surface properties of the polyaniline films, significantly influencing cell proliferation and migration; hence, the potential for application in the biomedical sector is limited, but opens the door for utilization as a biosensor. The surface energy constitutes the crucial factor that influences cell/surface interaction and the determination of surface energy is essential to attaining appropriate surface modifications.

## Figures and Tables

**Table 1:** Surface energy evaluation of different polyaniline surfaces.

Sample	Surface Energy Components (mN·m^−1^)			
	γ^tot^	γ^LW^	γ^AB^	γ^dif^
PANI-S	52.54 *	46.05 *	6.49 *	3.33
PANI-B	50.88 *	46.54 *	4.35 *	1.67
PANI-SULF	52.13	44.97	7.17	2.92
PANI-PT	51.89	47.39	4.50	2.68
PANI-PAMPSA-1:1	41.85	40.98	0.87	7.36
PANI-PAMPSA-2:1	56.35	43.91	12.45	7.14
Cells	49.21	23.21	26.00	-

* The values presented in [14].
